# Differential diagnosis and identification of prognostic markers for peripheral T-cell lymphoma subtypes based on flow cytometry immunophenotype profiles

**DOI:** 10.3389/fimmu.2022.1008695

**Published:** 2022-11-18

**Authors:** Qiyao Pu, Jie Qiao, Yuke Liu, Xueyan Cao, Ran Tan, Dongyao Yan, Xiaoqian Wang, Jiwei Li, Baohong Yue

**Affiliations:** ^1^ Department of Laboratory Medicine, the First Affiliated Hospital of Zhengzhou University, Zhengzhou, Henan, China; ^2^ Key Clinical Laboratory of Henan Province, Zhengzhou, Henan, China; ^3^ School of Basic Medical Sciences, Zhengzhou University, Zhengzhou, Henan, China; ^4^ Department of Oncology, the First Affiliated Hospital of Zhengzhou University, Zhengzhou, Henan, China; ^5^ Faculty of Laboratory Medicine, Zhengzhou University, Zhengzhou, Henan, China

**Keywords:** peripheral T-cell lymphoma, FCM immunophenotype, AITCL, T-CUS, differential diagnosis, prognostic marker, targeted therapy

## Abstract

We compared the differential expression of 15 markers in PTCL (Peripheral T-cell lymphoma) subtypes and T-CUS (T-cell clones of uncertain significance), and summarized the specific immunophenotype profiles of each subtype and its impact on prognosis. PD-1 and CD10 are diagnostic markers for AITL (angioimmunoblastic T-cell lymphoma). To avoid confusion with T-CUS of benign clones, it is recommended to define AITL as bounded by PD-1+%>38.01 and/or CD10+%>7.46. T cell-derived ENKTL-N (extranodal NKT cell lymphoma) specifically expresses CD56. ALCL (anaplastic large cell lymphoma) characteristically expresses CD30 and HLA-DR. PTCL-NOS (peripheral T-cell lymphoma unspecified) still lacks a relatively specific phenotype and is prone to loss of basic lineage markers CD3, CD5, and CD7. The determination of T-CUS can be verified by the overall assessment of the bone marrow and a certain period of follow-up. The clustering results showed that the expression of 8 specific markers was significantly different among the 5 groups, suggesting that a combination of related markers can be analyzed in the identification of PTCLs subtypes. The study explores the advantages of TRBC1 combined with CD45RA/CD45RO in detecting T cell clonality, which can efficiently and sensitively analyze multiple target T cell populations at the same time. The sensitivity of PB to replace BM to monitor the tumor burden or MRD (minimal residual disease) of PTCLs is as high as 85.71%, which can relieve the huge pressure of clinical sampling and improve patient compliance. CD7, CD38, and Ki-67 are prognostic indicators for AITL. CD3 and CD8 on PTCL-NOS, and CD56 and HLA-DR on ENKTL-N have prognostic role. This study supports and validates the current classification of PTCL subtypes and establishes an immunophenotypic profile that can be used for precise diagnosis. The important clinical value of PTCLs immunophenotype in routine classification diagnosis, clonality confirmation, prognosis prediction, and treatment target selection was emphasized.

## Introduction

Peripheral T-cell lymphomas (PTCLs) are a group of rare mature T-lymphocytic malignancies with a relatively high incidence in Asia, accounting for about 25%-30% of NHL, significantly higher than those in Europe and The clustering results showed that the expression of 8 specific markers was significantly different among the 5 groups the United States (5%-10%) (1, 2). Most PTCLs are highly aggressive, highly malignant, and have a poor prognosis. Bone marrow infiltration often occurs in the middle and late stages. PTCLs have a wide range of clinical behaviors, with the significant group and individual heterogeneity in terms of disease progression, biological behavior, treatment effects, and prognosis and survival (3, 4). The 5-year survival rate for PTCLs is only around 30%, which contrasts with recent advances in Hodgkin lymphoma and mature B-cell lymphoma (5–7). A small subset of PTCLs, such as anaplastic large cell lymphoma (ALCL), have a relatively good prognosis (8). However, most cases diagnosed with PTCLs eventually die from the disease.

The extremely low survival rate correlates with the degree of difficulty in diagnosing the disease when conventional morphological and immunohistochemical means are used. Classification of PTCLs involves the division of multiple subtypes, which are often low-frequency and ambiguous. Accurate diagnosis of PTCLs becomes even more important with the advent of novel treatment options with subtype-specificity (9–11). Accurate identification of PTCLs subtypes can help formulate appropriate treatment plans and help clinically grasp disease outcomes. With the development of novel antibody and clonality assessment methods, flow cytometry has become one of the main diagnostic tools for hematological malignancies. The broad immunophenotypic and morphological spectrum easily overlaps with T-cell clones of uncertain significance (T-CUS), making the identification of PTCL subtypes difficult (12). Therefore, comprehensive immunophenotype needs to be monitored when diagnosing subclasses of PTCLs.

This study attempts to identify a set of reliable and reproducible markers for accurate diagnosis and prognosis, which will help to achieve individualized clinical management of patients. We retrospectively analyzed and summarized the FCM immunophenotypic characteristics of a range of PTCL subtypes to distinguish malignant T cells from benign clones of T-CUS. Since there is an unmet need for reliable prognostic markers in PTCLs, this study explored the impact of different phenotypes on the prognosis of PTCLs. We analyzed the relationship between 15 immune markers and the overall survival (OS) and progression-free survival (PFS) of each subtype of PTCLs, and screened out CD7, CD38, Ki-67, CD3, CD8, CD56, and HLA- DR as the prognostic value in different subtypes of PTCLs. In conclusion, we demonstrate the comprehensive clinical value of FCM immunophenotype in accurate diagnosis, the guidance of treatment, and the prognosis of PTCLs.

## Subjects and methods

### Patients and sample selection

**Figure 1:**
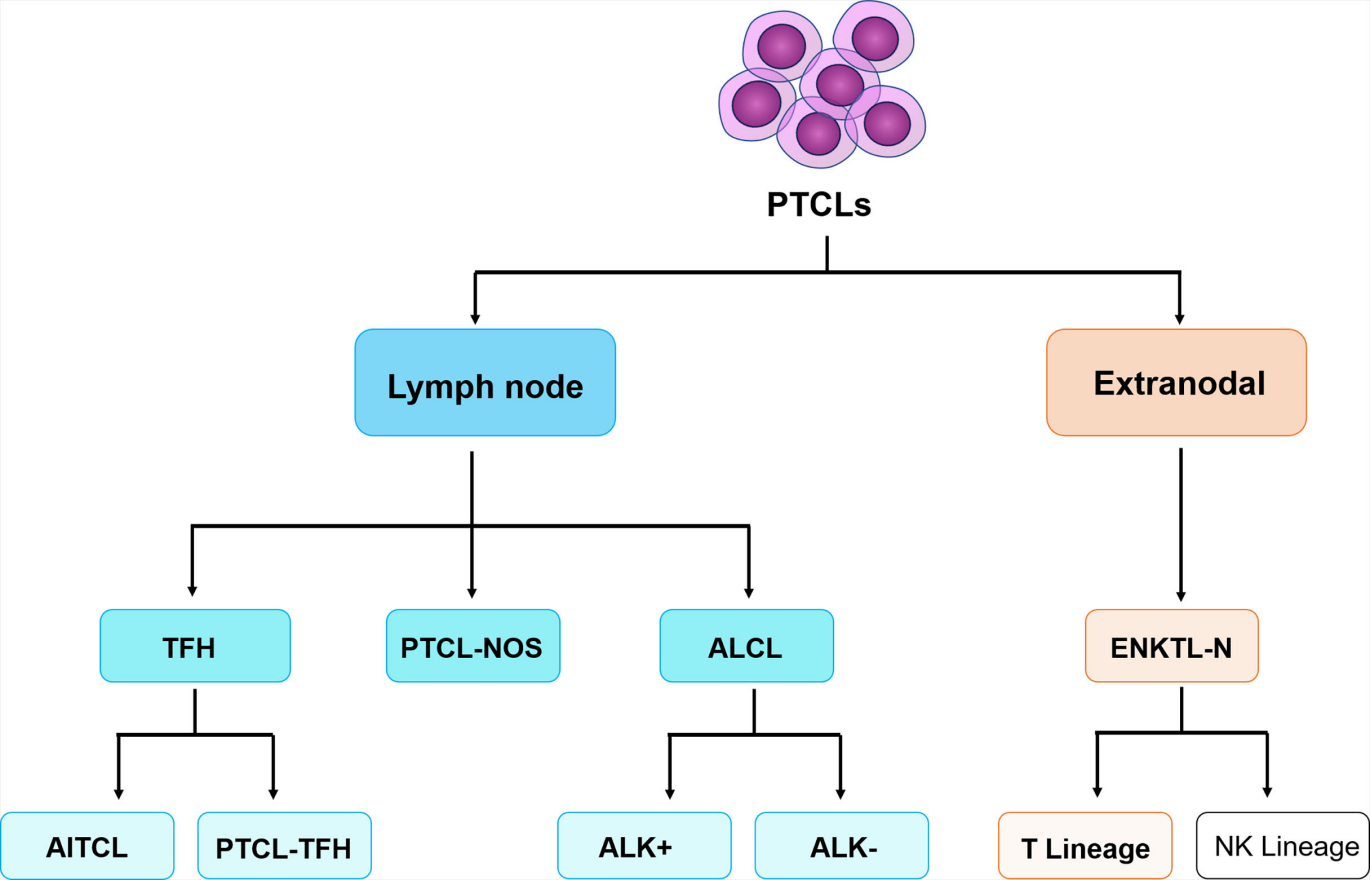
The classification of peripheral T-cell lymphomas follows the World Health Organization 2017 classification of lymphomas. PTCLs, Peripheral T-cell lymphomas. TFH, follicular helper T-cell; ALK, anaplastic lymphoma kinase; AITL, angioimmunoblastic T-cell lymphoma; PTCL-TFH, follicular helper T-cell lymphoma; PTCL-NOS, peripheral T-cell lymphoma tumor, unspecified; ALCL, anaplastic large cell lymphoma; ENKTL-N, extranodal NKT cell lymphoma. (Only the types included in this study are limited here).

Complete clinical data of peripheral T-cell lymphoma patients diagnosed and treated by the Lymphoma Center of the First Affiliated Hospital of Zhengzhou University from October 2020 to May 2022 were collected, including clinical course records, laboratory test results, pathological results, and imaging reports, etc. A total of 81 patients diagnosed with peripheral T-cell lymphoma with bone marrow infiltration and confirmed to participate in this study and 15 T-CUS patients with non-T-cell tumor-related diseases were included. According to the 2017 World Health Organization guidelines for diagnosis and treatment **(** **Figure 1** **)**, it is classified as angioimmunoblastic T-cell lymphoma (AITL, n=40), peripheral T-cell lymphoma unspecified (PTCL-NOS, n=25), extranodal NKT cell lymphoma (ENKTL, n=11), anaplastic large cell lymphoma (ALCL, n=5), and T-cell clones of uncertain significance (T-CUS, n=15). FCM Immunophenotype results from fresh peripheral blood or bone marrow aspirate samples from patients were retrospectively analyzed.

### Flow cytometry sample preparation

Bone marrow and peripheral blood samples were collected with K_2_-EDTA anticoagulant, and mature red blood cells were lysed within 24 h using 2 ml of 1x Lysing Buffer (BD Biosciences, San Jose, CA, USA) without fixative. The split cell suspension was maintained in PRMI1640 containing 5% fetal bovine serum and 5% dual antibiotics (penicillin and streptomycin). Divide the cell suspension into 3 test tubes, and the number of nucleated cells in each tube does not exceed 1×10^6^. Cells were stained with the following monoclonal antibodies correspondingly distributed in 3 tubes: FITC-conjugated CD57, PE-conjugated CD8, PerCP-conjugated CD3, APC-conjugated TRBC1, APCCy7-conjugated CD4, V450-conjugated CD7, V500-conjugated CD45, BV605-conjugated CD279; FITC-conjugated CD3, PE-conjugated CD2, PerCP-conjugated CD5, PE-Cy7-conjugated CD56, APC-conjugated CD10, APCCy7-conjugated CD16, V450-conjugated CD7, V500-conjugated CD45; BV605-conjugated CD4; FITC-conjugated Ki-67, PE-conjugated CD30, PerCP-conjugated CD3, PE-Cy7-conjugated CD56, APC-conjugated HLA-DR, APCCy7-conjugated CD20, V450-conjugated CD7, V500-conjugated CD45, and BV605-conjugated CD38. Among them, the (3) tube needs to be subjected to membrane permeation treatment. Detailed antibody information can be found in **Table S1**. After staining, samples were incubated at room temperature for 15 minutes in the dark, then treated with 0.009% sodium azide (AZ) and 0.07% ethylenediaminetetraacetic acid (EDTA) in 2 ml phosphate buffered saline (PBS) After two consecutive washing steps, centrifugation was performed at 500 × g for 5 min before samples were collected on a FACSCanto II cytometer (BD Biosciences, San Jose, CA, USA) using BD FACSDiva v1.6 software.

### Flow cytometry data analysis

Lymphocytes and monocytes were gated using side scatter (SSC) vs CD45 dot plots after exclusion of diploids and debris. Normal T cells are gated on the expression of CD3, T cells are defined as CD45+CD3+ events, and can be divided into CD4+ T cells and CD8+ T cells by CD4 and CD8. NK cells were gated on the expression of CD56 and the absence of CD3, and NK cells were defined as CD45+CD3-CD56+ events. For each group of cases (AITL, PTCL-NOS, ENKTL-N, ALCL, and T-CUS), the expression pattern of each marker of the clonal T cells studied were assessed. Using normal T cells and NK cells as internal controls, the fluorescence intensities of various intracellular and extracellular markers of clonal T cells were compared using a scatter plot. Data were analyzed and interpreted using Kaluza2.1 (Beckman Coulter, Brea, CA, USA) and Flowjo10.6.2 (BD Biosciences, San Jose, CA, USA).

### Statistical methods

For data meeting a normal distribution, two-sided unpaired t-tests were used to analyze continuous variables between the two groups. Pearson’s correlation coefficient was used to assess the correlation between the markers. Survival analysis included assessment of overall survival (OS) and progression-free survival (PFS), calculated using the Kaplan-Meier method. Cox proportional hazards models were used for univariate and multivariate analyses of prognostic markers, with hazard ratios (HR) and 95% confidence intervals (95% CI) reported. P<0.05 was statistically significant (*P<0.05, **P<0.01, ***P<0.001, ****P<0.0001). Data were processed and analyzed using GraphPad Prism 9.0(San Diego, CA, USA), SPSS Version 26.0 (SPSS, Chicago, IL), and R4.0.3 (R Foundation, Vienna, Austria).

## Results

### Basic characteristics of subjects

A total of 96 patients were included in this study, including 81 patients with PTCLs and 15 patients with T-CUS. The mean age of patients with PTCLs was 57.1 years (range: 13-80 years), and the proportion of male patients was higher (56.79%). The four subtypes of PTCLs were all prone to symptoms of superficial lymphadenopathy (72.84%), and ENKTL-N had the lowest proportion of this symptom (36.36%). Concomitant symptoms of anemia and liver dysfunction, the two most common complications in AITL and ALCL, respectively, were reported in 51 and 42 patients, respectively. There were 22 (22.92%) and 44 (54.32%) patients, respectively, with decreased absolute numbers of neutrophils and lymphocytes. Forty-five patients (55.56%) had serum LDH levels greater than 250 U/L. T-CUS patients were prone to anemia symptoms (73.33%) and elevated serum β2-MG levels (73.33%) due to their primary disease. The clinical characteristics of each subtype of PTCLs are detailed in **Table S2**. Most of the cases included in this study initially exercised the CHOP, CHOP-like, and CHOEP regimens. The mean follow-up time was 23.4 months (range: 3-85 months).

### Classification and diagnosis of PTCLs based on FCM immunophenotype

**Figure 2:**
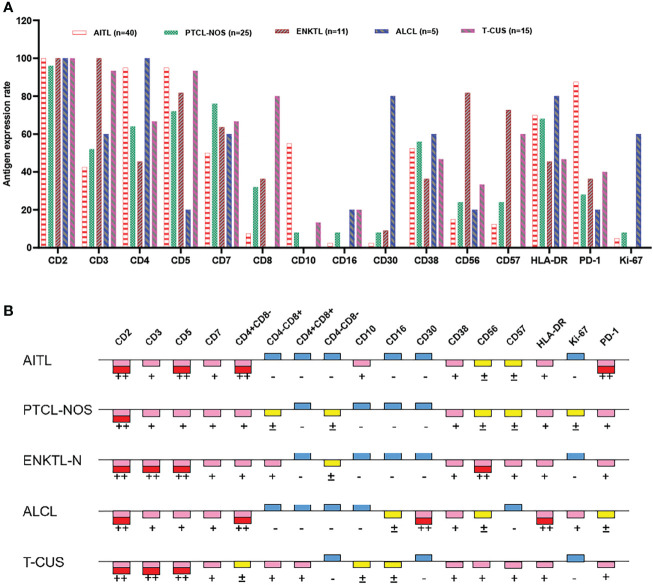
Expression profiles of immune markers for AITL, PTCL-NOS, ENKTL, ALCL, and T-CUS. **(A)** Proportion of positive expression of each antigen of 4 PTCL subtypes and T-CUS. **(B)** Antigen expression profiles of 4 PTCLs subtypes and T-CUS. ++, >75% of cases express this antigen; +, 25%-75% of cases express this antigen; ±, 10%-25% of cases express this antigen; -, <10% of cases express this antigen.

Clonal T cell populations were defined as immunophenotypically abnormal cell clusters that met one or more of the following criteria (1): absence or reduced expression of highly stable T lineage antigens (CD2/CD3) (2); Decreased expression of multiple lineage-associated antigens and altered light scattering properties (3); Mature T cells show monoclonality. **Figure 2** summarizes the antigen expression profiles of different subtypes of peripheral T-cell lymphoma. Details of the expression ratio of each marker in the five groups can be seen in **Table S3**. The expression of CD2 was stable in the PTCL subtype, and the expression rate was almost 100% in the 4 subtypes and T-CUS (only one case was lost in PTCL-NOS). AITL, PTCL-NOS, and ALCL are prone to loss of CD3 (loss rates are 57.50%, 48.00%, and 40.00%, respectively); ALCL is extremely prone to loss of CD5 (loss rate is 80%); The loss rates of CD7 in AITL, ALCL, ENKTL-N, and T-CUS were 50%, 40%, 36.36% and 33.33, respectively. Most clonal T cells in PTCL are CD4 positive, and CD8 positive is rare. In contrast, T-CUS cells were mostly CD8 positive (CD8+/CD4−: 46.67%, CD4−/CD8−: 0%, CD4+/CD8+: 33.33%, CD4+/CD8−: 20%). PD-1 (87.5%) and CD10 (55.0%) can be used as characteristic markers of AITL. 81.82% of ENKTL-N expressed CD56, while the expression rates of other subtypes of PTCLs were all below 30%. Except for the high expression of CD30 (80.0%) and Ki-67 (60.0%) in ALCL, other subtypes are hardly expressed.

**Figure 3:**
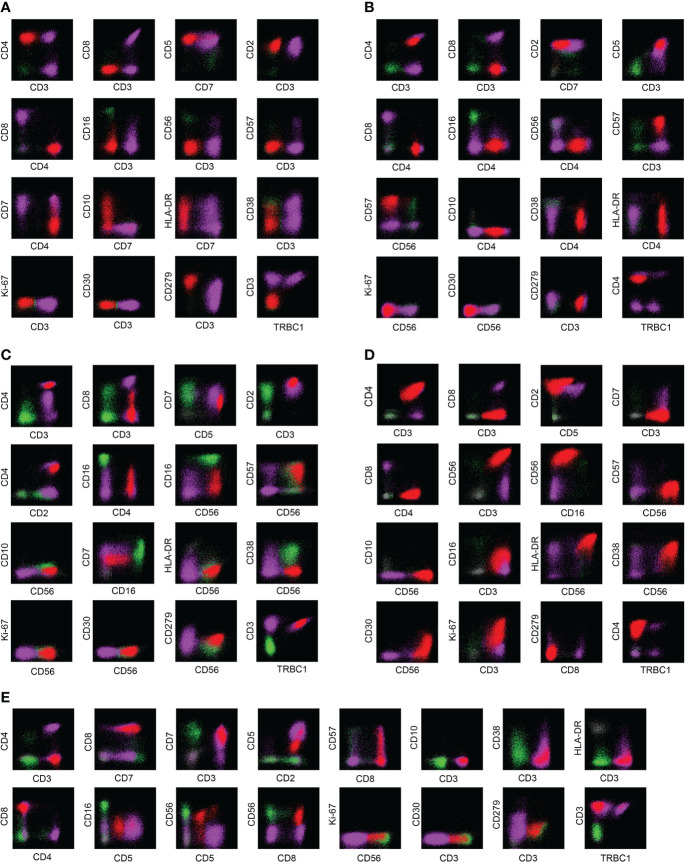
Examples of immunophenotypes for AITL, PTCL-NOS, ENKTL-N, ALCL, and T-CUS. **(A)** Typical FCM immunophenotype of AITL: CD3(-)CD4(+)CD10(+)PD-1(+); **(B)** Typical FCM immunophenotype of PTCL-NOS: CD2(+)CD4(+) CD7(-); **(C)** Typical FCM immunophenotype of ENKTL-N: CD3(+)CD56(+); **(D)** Typical FCM immunophenotype of ALCL: CD30(+)Ki-67(+); **(E)** Typical immunophenotype of T-CUS: CD3(+)CD8(+). The red dots in the figure are clonal T cells, the purple dots are background normal T cells, and the green dots are NK cells as an internal control.

The main features of AITL are: sCD3(-/dim), CD4(+), CD10(dim/+), PD-1(+); the main features of ENKTL-N are: sCD3(+), CD56(+); The main features of ALCL are: sCD3(-/dim), CD4(+), CD5 (–), CD30(+), HLA-DR(+). PTCL-NOS lacked characteristic marker expression, and often showed loss of expression of basal lineage markers CD3, CD5, and CD7 (loss rates were 48%, 28%, and 24%) **(** **Figure 3B** **)**. Similarly, the phenotypic distribution of T-CUS is highly heterogeneous, which is related to the status of the patient’s primary disease. The proportion of tumorous T cells losing CD3 and CD5 in PTCLs was 45.68% (37/81) and 18.52% (15/81), respectively. In contrast, the expression of CD3 and CD5 in T-CUS was relatively stable, and the loss rate of both is 6.67% (1/15). Therefore, the loss of these two lineage markers is likely to point to T-cell tumors, and the diagnosis of T-cell tumors should be cautious when CD3 and/or CD5 are expressed. **Figure 3** shows the FCM immunophenotype of typical cases of AITL, PTCL-NOS, ENKTL-N, ALCL, and T-CUS in this study. **Table 1** summarizes the typical immunophenotypic characteristics of the five test groups.

**Table 1:** Summary of typical immunophenotypic characteristics of AITL, PTCL-NOS, ENKTL, ALCL and T-CUS.

	CD2	CD3	CD4	CD5	CD7	CD8	CD10	CD16	CD30	CD38	CD56	CD57	HLA-DR	PD-1	Ki-67
AITL	+	+/-	+	+	+/-	–	+/-	–	–	+/-	-/+	-/+	+/-	+	–
PTCL-NOS	+	+/-	+/-	+/-	+/-	-/+	–	–	–	+/-	-/+	-/+	+/-	+/-	-/+
ENKTL-N	+	+	+/-	+	+/-	-/+	–	–	–	+/-	+	+/-	+/-	+/-	–
ALCL	+	+/-	+	+/-	+/-	–	–	-/+	+	+/-	-/+	–	+	-/+	+/-
T-CUS	+	+	-/+	+	+/-	+/-	-/+	-/+	–	+/-	+/-	+/-	+/-	+/-	–

### Cluster analysis of PTCLs and T-CUS based on immunophenotype

**Figure 4:**
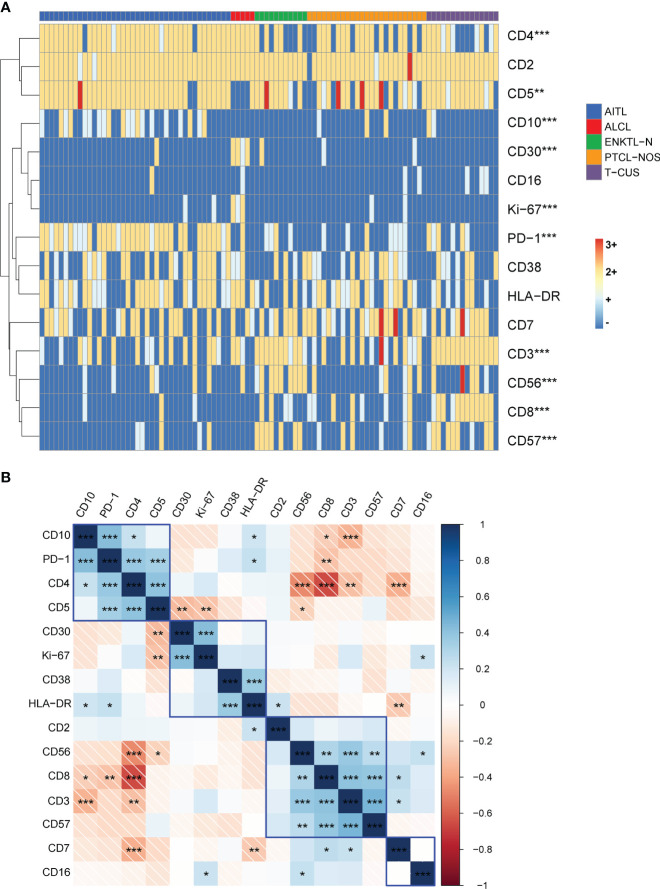
Immunophenotype-based cluster analysis of AITL, PTCL-NOS, ENKTL-N, ALCL, and T-CUS. **(A)** 15 immune markers of clonal T cells from patients with AITL (n=40), PTCL-NOS (n=25), ENKTL-N (n=11), ALCL (n=5), and T-CUS (n=15) clustering diagram. The horizontal axis represents individual patients, and the vertical axis represents immune markers. **(B)** Correlation matrix of immune markers of PTCLs. Pearson correlation matrix analyzes all possible correlations among 15 immune markers. (*p < 0.05, **p < 0.01, ***p < 0.001).

To explore the effect of multicolor flow cytometry in differentiating peripheral T-cell lymphoma subtypes, we performed an unsupervised cluster analysis of the subjects’ immunophenotype results, and the results are shown in **Figure 4**. As can be seen, the cluster plot clearly shows that the FCM phenotype is very effective in distinguishing disease groups. The Kruskal-Wallis rank sum test was further used to conduct multiple comparisons to analyze the expression differences of each marker among the four subtypes and T-CUS groups. We found that the expressions of CD3, CD4, CD5, CD8, CD10, CD30, CD56, and CD57 were significantly different among the five groups, while the expression distributions of CD2, CD7, CD16, CD38, and HLA-DR among the groups were not variability.

Then, all possible correlation coefficients between the 15 markers were analyzed using the Pearson correlation matrix, and we summarize the representative correlations as follows (1). some indicators express a positive correlation. There was a positive correlation between CD10, PD-1, CD4, and CD5; there was a positive correlation between CD30, CD38, HLA-DR, and Ki-67; there was a positive correlation between the expressions of CD2, sCD3, CD8, CD56, and CD57 (2). There are negatively correlated indicators of expression. CD4 and CD8, CD56; CD7 and CD2, CD5; sCD3 and CD10. We briefly explain it as follows. For AITL that most often express the two characteristic indicators of PD-1 and CD10, it is usually CD4-positive clonal T cells, and CD5 is not easily lost; CD30, CD38, and HLA-DR are positively correlated with the expression of indicators reflecting clonal T cell activation and Ki-67, which reflects proliferation ability; CD8-positive clonal T cells are prone to express CD56 and CD57, and the expression of CD2 is stable. However, CD4-positive clonal T cells are not easy to express CD56; clonal T cells stably express CD2 and CD5, but the loss rate of CD7 is high, and the loss rate of the cases included in this study is as high as 24%-50%; CD10-expressing clonal T cells frequently experience loss of CD3. Therefore, the above-mentioned results can be used to combine the relevant antibodies in the design of the antibody combination panel to explore their expression patterns.

### Immunophenotypic characteristics of T-CUS

There is a class of benign clonal T cells, which we tend to interpret as T-CUS given the lack of other clinical manifestations and laboratory findings to support the diagnosis of malignant T cell tumors. Its inclusion meets the following two principles (1): non-primary T cell tumors (2); mature T cell subsets are monoclonal. The majority of T-CUS can be traced back to the primary disease. The primary diseases of T-CUS included in this study were: 8 cases of B-cell lymphoma (4 cases of FL; 3 cases of DLBCL; 1 case of MZL), 1 case of MDS, 1 case of MM, 2 cases of anemia, 1 case of vasculitis, and 1 case of primary disease Solid tumor, 1 case of cerebral infarction.

Summary of the immunophenotype of 15 T-CUS cases. The loss rates of CD3, CD5 and CD7 were 6.67% (1/15), 6.67% (1/15) and 33.33% (5/15), respectively. 10 cases (66.67%) expressed or weakly expressed CD4, and 12 cases (80.00%) expressed or weakly expressed CD8. There were 2 cases (13.33%) of T-CUS expressing CD10, both of which were patients with primary FL. Six patients (40%) expressed or partially expressed PD-1. The proportions of expressing CD38 and HLA-DR were 46.67% (7/15) and 46.67% (7/15), respectively. The expression rates of NK lineage-related markers CD16 and CD56 were 20.00% (3/15) and 33.33% (5/15), respectively. The expression rate of CD57 was (9/15). CD30 and Ki-67 were not expressed in any case. With a certain period of follow-up, T-CUS tends to disappear within a few months.

### CD3(-/dim) CD4(+) cell subsets combined with PD-1 and CD10 can screen and diagnose AITL

**Figure 5:**
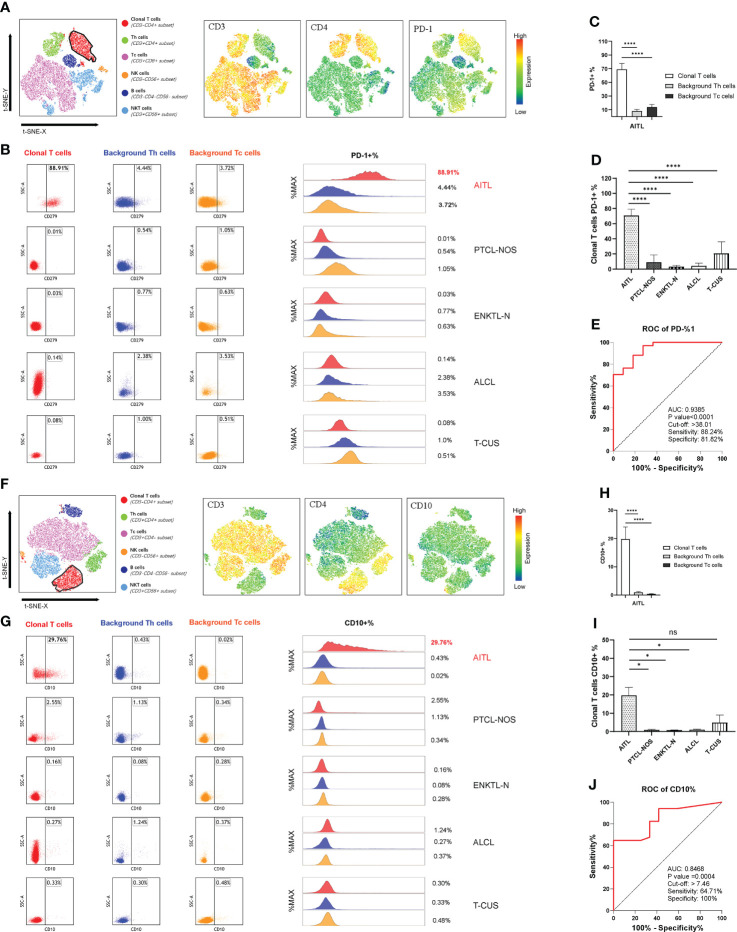
PD-1 and CD10 specificity patterns of AITL. **(A)** t-SNE map of AITL patients showing the expression pattern of CD3-CD4+PD-1+. Different colors represent different cell clusters. The positions of the red clusters are clonal T cells of AITL. **(B)** PD-1 expression in AITL, PTCL-NOS, ENKTL-N, ALCL and T-CUS cloned T cells and background normal Th and Tc cells. **(C)** Histogram of mean PD-1 expression rates in clonal T cells and background normal Th and Tc cells in patients with AITL. **(D)** Histograms of mean PD-1 expression rates on clonal T cells in patients with AITL, PTCL-NOS, ENKTL-N, ALCL, and T-CUS. **(E)** Receiver operating characteristic (ROC) curves evaluating PD-1-defined AITL. **(F)** t-SNE plot of AITL patients showing the expression pattern of CD3-CD4+CD10+. Different colors represent different cell clusters. The positions of the red clusters are clonal T cells of AITL. **(G)** CD10 expression of AITL, PTCL-NOS, ENKTL-N, ALCL and T-CUS cloned T cells and background normal Th and Tc cells. **(H)** Histogram of the average expression rate of CD10 in clonal T cells and background normal Th and Tc cells in patients with AITL. **(I)** Histogram of mean CD10 expression rates in clonal T cells in patients with AITL, PTCL-NOS, ENKTL-N, ALCL and T-CUS. **(J)** Receiver operating characteristic (ROC) curves evaluating CD10-defined AITL. (*p < 0.05, ****p < 0.0001)

Most of the clonal T cells in PTCLs expressed CD4 (64/81; 79.01%), and CD8 expression was rare (15/81; 18.52%). Among them, the proportion of CD4-positive clonal T cells that lost CD3 or weakly expressed CD3 was 65.63% (42/64), and the phenomenon in AITL could be as high as 70% (28/40). The positive rate of PD-1 was 100% in sCD3-CD4+ AITL and 90% in total AITL. The positive rate of CD10 in sCD3-CD4+ AITL is not high, only 30%, but the positive rate in total AITL can be as high as 50%. **Figures 5A**, **5F** show the common expression characteristics of PD-1 and CD10 in AITL cells of the sCD3-CD4+ subset, respectively. The expression of PD-1 and CD10 in AITL cells was comprehensively compared horizontally and vertically. Both in the comparison of the clonal T cells of each subtype of PTCLs and the comparison with the autologous background T cells, the characteristics of significantly high expression were shown **(** **Figures 5B** **, D** **,** **G** **–J** **).**

The average expression rate of PD-1 in AITL cells was 68.95%, and the average expression rate of CD10 was 19.80%. T-CUS shows PD-1-positive clonal T cells due to the proliferation of most CD8-positive reactive T cells or CD10-positive benign clonal T cells due to some primary follicular lymphomas and marginal lymphomas. Therefore, it is necessary to formulate the cut-off value of the positive expression rate of PD-1 and CD10 to distinguish AITL from T-CUS. AITL could be effectively recognized when PD-1+%>38.01 or CD10+%>7.46 **(** **Figures 5E** **,** **I** **).** Its sensitivity and specificity were 88.24%, 81.82%, and 64.71%, 100%, respectively. The lower the value below these critical values, the more blurred the demarcation from T-CUS. The PD-1 bright+ CD10+ T cell population can effectively identify other PTCLs subtypes or T-CUS with similar morphology to AITL with high sensitivity and specificity. Therefore, the organic combination of CD3(-/dim) CD4+ with PD-1 and CD10 can comprehensively screen and diagnose AITL cells.

### Analysis of target T cell subset clonality by TRBC1 and CD45RA/CD45RO

**Figure 6:**
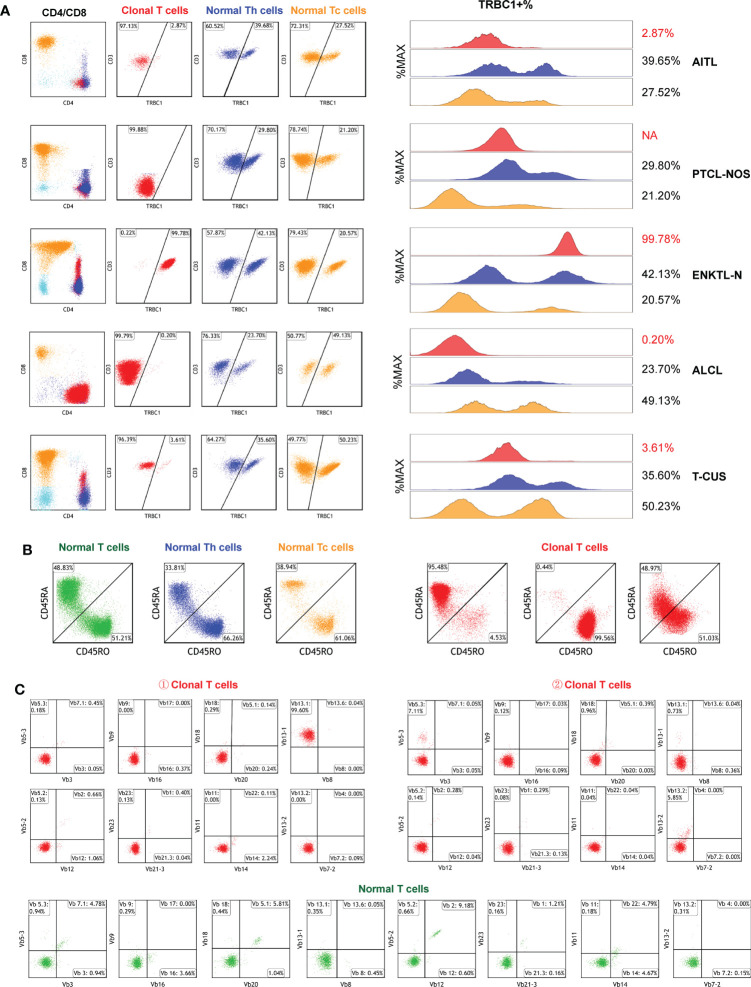
Clonal detection of T cells. **(A)** The clonality of each target T cell subset was detected by TRBC1. Red scatter points represent clonal T cells, blue scatter points represent background normal Th cells, and yellow scatter points represent background normal Tc cells. **(B)** Identification of the clonality of each target T cell subset by examining the CD45RA/CD45RO differentiation trajectories. Under normal circumstances, the differentiation trajectories of T cells are scattered and continuous, and under abnormal circumstances, they tend to aggregate into clusters. **(C)** Validation of T cell subset clonality by TCR-vβ immunophenotype distribution. Clonal T cells are restricted to a single TCRVβ subset (more than 40–50%), or lack expression.

We investigated TRBC1 expression patterns in abnormal T cells and background normal T cells in 96 subjects. The expression of TRBC1 in clonal T cells was monoline-type and divided into two patterns: the positive rate of TRBC1 was >85% and <15%. Of all the CD3-positive subjects included, 19 (32.76%) monoclonal T cells had positive TRBC1 expression, and 39 (67.24%) monoclonal T cells had negative TRBC1 expression. The expression pattern of TRBC1 in normal T cells is polymorphic. Both background normal CD4+ T cells and CD8+ T cells showed scattered expression of TRBC1 in T-cell tumors **(** **Figure 6A** **)**.

TRBC1 does not work on CD3- cells, so in 38 subjects with CD3 loss (CD3-negative as shown in **Figure 6A** **-**PTCL-NOS) we further assessed clonality by judging the differentiation trajectories of CD45RA and CD45RO. The differentiation trajectories of neoplastic T cells were significantly different from those of normal T cells, and their abnormal differentiation was mainly reflected in three aspects (**Figure 6B**). They were: ① Clonal T cells expressed CD45RA intensively, which was detected in 50.00% (19/38) of subjects; ② Clonal T cells expressed CD45RO intensively, which was found in 42.11% (16/38) of patients; and ③ CD45RA and CD45RO were co-expressed in clonal T cells, but they were clustered and their differentiation trajectories were ambiguous and were detected in only 3 subjects (7.89%).

To verify the effect of TRBC1 and CD45RA/CD45RO as indicators of T cell clonality, we randomly analyzed the TCR-Vβ profiles of peripheral blood samples from 10 patients with clonal T cell subsets **(** **Figure 6C** **)**. TCR-Vβ-restricted expression was shown in all cell populations examined, confirming the clonality of T cells **(** **Table S4** **)**. The combination of TRBC1 and CD45RA/CD45RO enables a rapid, simple, and highly sensitive comprehensive analysis of the clonality of target T cell subsets.

### Blood monitoring of circulating tumor cells after PTCLs treatment

**Figure 7:**
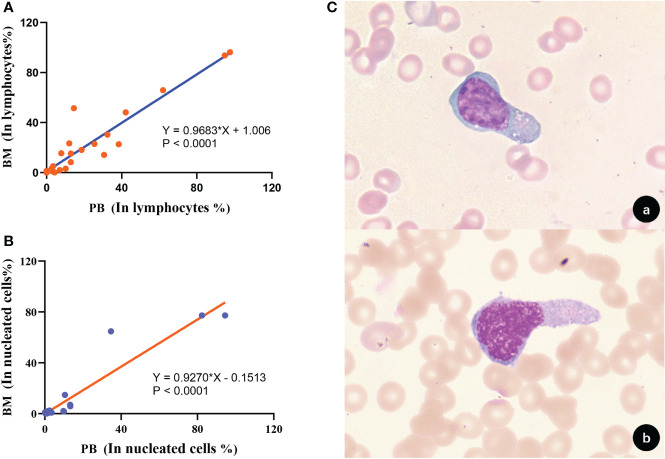
Correlation and homology of clonal T cell burden in BM and PB. **(A)** The proportion of clonal T cells to lymphocytes in BM and PB showed a positive correlation (p<0.0001). **(B)** The proportion of clonal T cells occupied nucleated cells in BM and PB showed a positive correlation (p<0.0001). **(C)** Morphological analysis confirmed that tumor T cells in BM and PB had consistent morphological features [**(A)**, BM; **(B)**, PB; 1000×].

We followed up and collected 28×2 peripheral T-cell lymphoma marrow-infiltrating paired BM and PB samples for tumor burden of circulating lymphoma cells by FCM. Based on obtaining 1 million effective cells and taking 20 events as the detection limit, circulating peripheral T lymphoma cells (CPTLs) could be detected in 85.71% (24/28) of the patients after treatment. The immunophenotype and clonal properties of tumor cells in paired samples were analyzed, and it was confirmed that the two had identical FCM characteristics. The morphology of CPTLs in peripheral blood and PTCLs in bone marrow were confirmed by cytomorphology **(** **Figure 7C** **)**. It was confirmed that the two were homologous cells. The proportion of circulating PTCLs cells and disseminated PTCLs cells occupying lymphocytes showed a positive correlation distribution, the r value was 0.9683, p<0.0001; the proportion of nucleated cells was also positively correlated, the r value was 0.9270, p<0.0001 **(** **Figures 7A, B** **)**. The regression fitting equation is shown in the figure. It can be seen that when disseminated lymphoma cells occupy more than 1.006% of lymphocytes, CPTLs can be detected by FCM. The detection sensitivity and specificity of CPTL were 85.71% and 100%, respectively.

### Immunophenotype-based prognostic significance of PTCLs

The overall 1-, 3-, and 5-year OS rates of the patients with PTCLs included in this study were 79.8%, 47.6%, and 28.6%, respectively, and the 1-, 3-, and 5-year PFS rates were 77.3%, 38.2%, and 14.3%, respectively. To explore prognostic markers in specific subtypes of PTCLs, we analyzed the effects of 15 markers on overall survival (OS) and progression-free survival (PFS) in AITL, PTCL-NOS, and ENKTL-N subtypes **(** **Tables S5, S6**).

**Figure 8:**
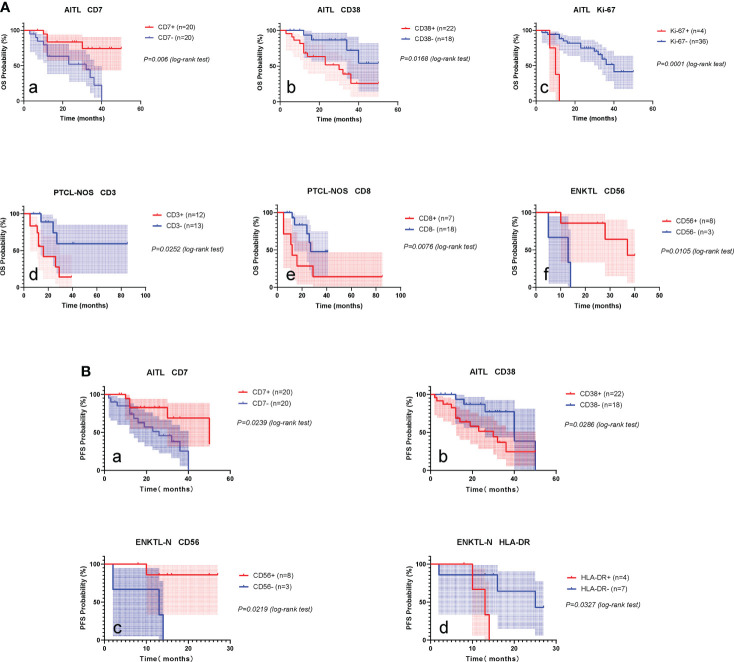
Prognostic markers of PTCLs. **(A)** Prognostic markers influencing OS of AITL, PTCL-NOS, ENKTL-N. **(a–c)**, OS survival curves of CD7, CD38 and Ki-67 on AITL. **(d, e)**, OS survival curves of CD3 and CD8 on PTCL-NOS. **(f)**, OS survival curve of CD56 versus ENKTL-N. **(B)** Prognostic markers influencing PFS in AITL and ENKTL-N. **(a, b),** PFS survival curves of CD7 and CD38 versus AITL. **(c, d)**, PFS survival curves of CD56 and HLA-DR against ENKTL-N.

The mean follow-up period for AITL was 24.0 months, the 2-year OS was 67.0% (51.5-82.5), and the 2-year PFS was 61.3% (44.3-78.4). Kaplan-Meier analysis showed that the expressions of CD7, CD38, and Ki-67 were significantly correlated with the OS of AITL **(** **Figures 8A** **, a-c** **)**. CD7 and CD38 correlated with the PFS of AITL **(** **Figures 8B** **, a-b** **)**. The COX univariate analysis confirmed that CD7 and CD38 had prognostic significance for AITL, and COX multivariate analysis indicated that CD7 and CD38 were independent prognostic factors for AITL (**Tables S7, S8**).

The mean follow-up period for the PTCL-NOS subtype was 22.0 months, the 2-year OS was 57.8% (34.7-80.9), and the 2-year PFS was 51.5% (25.43-77.57). Kaplan-Meier analysis showed that the expression of CD3 and CD8 was significantly correlated with the OS of PTCL-NOS **(** **Figures 8A** **, d, e** **)**. COX univariate analysis confirmed that CD3 and CD8 had prognostic significance for PTCL-NOS (**Table S9**).

The mean follow-up period for ENKTL-N was 22.2 months, the 2-year OS was 56.2% (29.5-82.9), and the 2-year PFS was 53.7% (26.1-81.3). Kaplan-Meier analysis showed that CD56 expression was significantly correlated with OS of ENKTL-N **(** **Figures 8A** **, f** **)**, and CD56 and HLA-DR had significant effects on PFS of ENKTL-N **(** **Figures 8B** **, c-d** **)**. COX univariate analysis determined that CD56, and HLA-DR had prognostic significance for ENKTL-N **(** **Tables S10, S11** **)**.

## Discussion

We retrospectively analyzed the FCM results of bone marrow and peripheral blood samples from a large number of patients with primary and relapsed peripheral T-cell lymphomas, including patients with confirmed PTCLs based on clinical findings such as histopathology and/or molecular biology.

In our laboratory routine, we use 3-tube 10-color MFC panels as the first diagnostic method for suspected T-cell lymphoproliferative disorders **(** **Table S1** **)**. We determined a total of 16+ (4) T-cell lymphoma-related markers, including lineage markers (CD2, CD3, CD4, CD5, CD7, CD8); NK lineage markers (CD16, CD56); follicular-derived markers CD10; T-cell activation markers CD30, CD38 and HLA-DR; terminal differentiation markers CD57; immune checkpoint PD-1; Ki-67 for judging tumor malignant proliferation ability; and judging T-cell clonal Marker TRBC1. For abnormal T cells with loss of sCD3, TCR markers (TCRαβ, TCRγδ) and differentiation markers (CD45RA, CD45RO) of T cells were additionally determined to further determine their clonal properties (13–15).

In this study, monitoring the immunophenotype of PTCLs in bone marrow infiltration has the following advantages (1): FCM can accurately observe the expression of multiple markers on target cell populations, and help identify disease subtypes by analyzing the specific phenotype of PTCLs (2);The FCM operation is relatively fast and simple, and can quickly obtain as many cells as possible in a short time, especially for the detection of low-frequency and trace PTCLs cells for comprehensive and efficient analysis (3); The four PTCLs subtype tumor cells were compared horizontally with T-CUS, and longitudinally with their background normal T cells.

The common abnormalities of peripheral T-cell lymphoma based on FCM can be summarized as follows (1): CD4/CD8 ratio imbalance, greater than 10:1 or less than 1:10 (2); abnormal expression of pan-T cell markers, CD3, CD5, and CD7 expression Abnormalities (attenuated, lost or up-regulated) are more common, and stable CD2 expression is relatively rare (3); abnormally elevated expression of certain markers in T cells (CD16, CD56, CD57, PD-1, etc.) (4); With abnormal marker expression (CD10, CD30, Ki-67) (5); Mature T cells appear monoclonal (restricted expression of TRBC1, CD45RA/CD45RO or TCR-vβ). For the identification of PTCL subtypes, CD4 and CD8 can be used as entry points, and PTCLs are more common in CD4+CD8-. AITL characteristically expresses follicular helper T cell (TFH)-related antigens CD10, and PD-1 (CD279), and does not express or weakly express sCD3 antigen. ALCL generally has larger FSCs and characteristically expresses CD30. Pan-T markers such as CD3, CD5, and CD7 are prone to deletion, often showing a “null cell” phenotype. ENKTL-N often expresses the NK lineage marker CD56. The immunophenotype of PTCL-NOS relatively lacks specificity, and it is often positive for CD4, and the expression of CD3, CD5, and/or CD7 is absent. It is necessary to exclude other types of PTCLs before diagnosis. Our results partially validate that there are consistent differences among the 4 major lymphoma subtypes, thus confirming the subclassification proposed by the World Health Organization in 2016, and confirmatory validation by the combined clinical results (16).

TRBC1 is an effective indicator for determining the clonality of TCRαβ+ T cells (14, 17–19), and about 95% of TCRαβ+ T cells are CD4+ or CD8+ T cells, so we combined TRBC1 with CD4 and CD8 when designing the panel. For the detection of malignant expansion of clonal T cells in PTCLs, previous TCR-Vβ analysis provided a method to detect the immunophenotype of TCR cells, but it was labor-intensive, expensive, and limited in sensitivity (20–22). The detection mode of TRBC1+CD45RA/CD45RO is more convenient and fast. However, in patients with non-T-cell tumors, small clonal subpopulations with deviating TRBC1 positivity rates are occasionally detected. In 2020, Min Shi et al. introduced the concept of T-cell clones of undetermined significance (T-CUS) due to the lack of other clinical or laboratory features supporting the diagnosis of T-cell malignancies (12). T cells, as immune cells, conduct targeted responses against antigenic determinants, generate an immune response, and further generate dominant clonal T cells. Clonal CD8+ T cells are more common than CD4+ T cells and have been associated with responses to common viral infections and tumors (23, 24). FCM appear as detectable monoclonal T cell populations. Therefore, in the application of TRBC1, more and more comprehensive T cell panels containing TRBC1 are needed to evaluate the analytical sensitivity. For the diagnosis of T lymphoma, the FCM phenotype requires a significant tumor burden (>30% tumor cells) and a detectable abnormal immunophenotype. In the absence of any other immunophenotypic abnormalities, the percentages should be interpreted with caution when they are at critical values. In addition, the determination of T cell clonality is a comprehensive process. Due to the particularity of TRBC1 in TCRαβ cells, it is not applicable to judge the clonality of T cell proliferation patients who do not express TCR or only express TCRγδ. When necessary, TCR gene molecular rearrangement detection and flow TCR-V β detection were combined for comprehensive diagnosis.

In recent years, the detection of circulating tumor cells has become a hot spot in the monitoring of various bone marrow diseases (25–27). When monitoring whether PTCLs have bone marrow infiltration and the degree of infiltration, CPTLs cells can be used instead of bone marrow PTCLs cells to screen and diagnose mature T-cell lymphomas to a certain extent. This is mainly due to the minimally invasive nature of blood compared to bone marrow; the absolute count of CPTLs is more accurate than the MRD of bone marrow due to possible bone marrow dilution; the observed correlation of tumor burden between the two. While the invasiveness of the bone marrow aspirate procedure limits frequent bone marrow sampling, longer-term follow-up of blood CPTLs is feasible and efficient, leading to improved patient compliance. Therefore, the detection of CPTLs in the blood can reflect the treatment effect and the degree of disease progression during the review after PTCLs bone marrow infiltration treatment.

To explore the impact of different immune markers on the prognosis of PTCLs, we analyzed the relationship between 15 immune markers (except TRBC1) and the overall survival (OS) and progression-free survival (PFS) of each subtype of PTCLs. It was found that, in AITL, patients expressing CD7 had a better prognosis, whereas patients expressing CD38 and Ki-67 had a poorer prognosis **(** **Figure 7** **)**, It may suggest that tumor cells with more active proliferative capacity are more aggressive. The expression of CD3 and CD8 in the PTCL-NOS subtype suggests a poor prognosis. Among the ENKTL-N subtypes, CD56-expressing patients had a favorable prognosis, whereas HLA-DR-expressing patients had significantly poorer survival outcomes. The expression of the above markers has important clinical value for the rapid clinical judgment of the prognosis of PTCLs and is expected to become a new clinical prognostic reference index. Whether the above immune markers can be used as independent prognostic factors of PTCLs still needs multi-regional research to verify.

Comprehensive immunophenotype results have very important clinical value in guiding targeted therapy of PTCLs (28–30). Antibody drugs targeting CD30 have achieved good efficacy in CD30+ PTCLs (31–33). Targeted CD38 antibody drugs also have a good response rate in the treatment of CD38-positive PTCLs (34, 35). For AITL and PTCLs with TFH phenotype, there have been several clinical trials to prove the efficacy of PD-1 inhibitors and PD-L1 (36, 37). Chimeric antigen receptor (CAR) T cell therapy, as a novel treatment for hematological malignancies, has also been tried in PTCLs. Clinical trials of CAR-T cells targeting CD7 and CD30 for the treatment of CD7-positive or CD30-positive T-NHL are being explored (38–41). At the same time, the combination of targeted drugs can further improve the prognosis of relapsed and refractory patients (42). Recent advances indicate that different molecular subtypes can be defined through the introduction of techniques such as gene expression profiling (GEP). They have obvious biological differences in carcinogenic pathways and prognosis. Precise subclassification can be used for patient management, including more appropriate and novel treatment protocols, which may facilitate the diagnostic application of new antibodies and the development of new therapeutic interventions. These include possible new diagnoses by flow cytometry and recently developed therapies. For example, the PTCL-GATA3 subtype may benefit from a chimeric anti-CCR4 monoclonal antibody that is effective against adult T-cell leukemia/lymphoma (43–45). In conclusion, accurate molecular subclassification of PTCL is essential for selecting the most appropriate diagnostic and therapeutic options for patients in future clinical trials.

In conclusion, this study systematically summarizes the immunophenotypic differential characteristics of 4 common PTCLs subtypes and T-CUS to provide a more specific diagnosis and prognosis. We discussed the importance of FCM in predicting prognosis and formulating individualized treatment. Establishing a more complete prognostic stratification system based on FCM immunophenotype has become the main goal of our current research.

## Data availability statement

The original contributions presented in the study are included in the article/**Supplementary Material**. Further inquiries can be directed to the corresponding author.

## Ethics statement

The studies involving human participants were reviewed and approved by The Ethics Committee of the First Affiliated Hospital of Zhengzhou University. The patients/participants provided their written informed consent to participate in this study.

## Author contributions

BY, QP, YL, JL designed the study. QP and XC performed experiments. QP and JQ performed data collection and analysis. QP, DY, XW and RT collected the clinical information and classified the patients. QP and JQ performed the statistical analysis and drafted. All authors contributed to the article and approved the submitted version.

## Funding

This work was supported by the Natural Science Foundation of Henan Province [grant numbers 162300410299]; the Henan Medical science and technology public relations project [grant numbers 2018020007; LHGJ20190038].

## Acknowledgments

We thank the subjects for agreeing to participate in this project. We thank clinical oncologists for their interest and participation in this study.

## Conflict of interest

The authors declare that the research was conducted in the absence of any commercial or financial relationships that could be construed as a potential conflict of interest.

## Publisher’s note

All claims expressed in this article are solely those of the authors and do not necessarily represent those of their affiliated organizations, or those of the publisher, the editors and the reviewers. Any product that may be evaluated in this article, or claim that may be made by its manufacturer, is not guaranteed or endorsed by the publisher.
