# Supplementary material for: Differential diagnosis and identification of prognostic markers for peripheral T-cell lymphoma subtypes based on flow cytometry immunophenotype profiles
Source: Front Immunol. 2022 Nov 18;13:1008695. doi: 10.3389/fimmu.2022.1008695 (PMC9715969; doi:10.3389/fimmu.2022.1008695)
Supplement: Supplementary file 1 [file DataSheet_1.docx]

| **Supplementary Material**  **Supplementary Tables**  Table S1: Antibody combination panel. | | | | | | |
| --- | --- | --- | --- | --- | --- | --- |
| *Tubes* | *Molecular target* | *Fluorescein* | *Function* | *Clone* | *Cat No* | *Manufacturers* |
| 1 | CD57 | FITC | Terminal differentiation | HNK-1 | 347393 | BD biosciences |
| 1 | CD8 | PE | Tc cells | SK1 | 340046 | BD biosciences |
| 1 | TRBC1 | APC | T cell clonality | JOVI.1 | STRBCAP04 | Caprico Biotechnologies |
| 1 | CD4 | APC-cy7 | Th cells | SK3 | 341105 | BD biosciences |
| 1 | PD-1 (CD279) | BV605 | Immune checkpoint | EH12.1 | 563245 | BD biosciences |
| 2 | CD3 | FITC | T cells | SK7 | 349201 | BD biosciences |
| 2 | CD2 | PE | Pan-T | S5.2 | 346597 | BD biosciences |
| 2 | CD5 | PerCP | Pan-T | L17F12 | 341099 | BD biosciences |
| 2 | CD10 | APC | Follicular source | HI10a | 340922 | BD biosciences |
| 2 | CD16 | APC-cy7 | NK cells | 3G8 | 557758 | BD biosciences |
| 2 | CD4 | BV605 | Th cells | SK3 | 566908 | BD biosciences |
| 3 | Ki-67 | FITC | Proliferation | 35/KI-67 | 612472 | BD biosciences |
| 3 | CD30 | PE | Activation | Ber-H83 | 341635 | BD biosciences |
| 3 | HLA-DR | APC | Activation | L243 | 340691 | BD biosciences |
| 3 | CD20 | APC-cy7 | B cells | L27 | 335812 | BD biosciences |
| 3 | CD38 | BV605 | Activation | HB7 | 663189 | BD biosciences |
| 4* | TCRαβ | FITC | Pan-TCRαβ | WT31 | 347773 | BD biosciences |
| 4* | TCRγδ | PE | Pan-TCRγδ | 11F2 | 340887 | BD biosciences |
| 4* | CD45RA | PE-CY7 | Differentiation | L48 | 649457 | BD biosciences |
| 4* | CD3 | APC | T cells | SK7 | 340661 | BD biosciences |
| 4* | CD4 | APC-cy7 | Th cells | SK3 | 341105 | BD biosciences |
| 4* | CD45RO | BV605 | Differentiation | UCHL1 | 562791 | BD biosciences |
| 1,3,4* | CD3 | PerCP | T cells | SK7 | 340663 | BD biosciences |
| 1,2,3 | CD56 | PE-cy7 | NK cells | B159 | 557747 | BD biosciences |
| 1,2,3 | CD7 | V450 | Pan-T | M-T701 | 642921 | BD biosciences |
| 1,2,3,4* | CD45 | V500 | Lymphocyte | HI30 | 560777 | BD biosciences |
| **: Optionally add this tube.* | | | | | | |

**Table S2: Clinical characteristics of patients.**

|  |  | *No. (n/N) (%)* | | | | |  |
| --- | --- | --- | --- | --- | --- | --- | --- |
|  | Overall PTCLs sample (n=81) | AITCL(n=40) | PTCL-NOS(n=25) | ENKTL (n=11) | ALCL(n=5) | T-CUS(n=15) |  |
|  |  |  |  |  |  |  |  |
| Age, years, mean (SD) | 57.1 | 60.36 | 57.24 | 51.55 | 49.8 | 64 |  |
| Male sex | 46 (56.79) | 24 (60.00) | 15 (60.00) | 6 (54.54) | 1 (20.00) | 10 (66.67) |  |
| Symptoms at onset of illness | | | | | | |  |
| Fever | 30 (37.04) | 21 (52.50) | 3 (12.00) | 5 (45.45) | 1 (20.00) | 3 (20.00) |  |
| Fatigue | 25 (30.86) | 17 (42.50) | 3 (12.00) | 3 (27.27) | 2 (40.00) | 5 (33.33) |  |
| lymphadenopathy | 59 (72.84) | 35 (87.50) | 16 (64.00) | 4 (36.36) | 4 (80.00) | 5 (33.33) |  |
| Splenomegaly | 42 (51.85) | 25 (62.50) | 9 (36.00) | 5 (45.45) | 3 (60.00) | 7 (46.67) |  |
| Complications | | | | | | |  |
| Anemia | 51 (62.96) | 30 (75.00) | 10 (40.00) | 8 (72.72) | 3 (60.00) | 11 (73.33) |  |
| Hyperlipidemia | 29 (35.80) | 14 (35.00) | 8 (32.00) | 5 (45.45) | 2 (40.00) | 3 (20.00) |  |
| Hypoproteinemia | 32 (39.51) | 20 (50.00) | 4 (16.00) | 6 (54.54) | 2 (40.00) | 7 (46.67) |  |
| Hepatic dysfunction | 42 (51.85) | 22 (55.00) | 9 (36.00) | 7 (63.63) | 4 (80.00) | 7 (46.67) |  |
| Renal dysfunction | 27 (33.33) | 10 (25.00) | 9 (36.00) | 6 (54.54) | 2 (40.00) | 4 (26.67) |  |
| Coagulation dysfunction | 27 (33.33) | 16 (40.00) | 5 (20.00) | 4 (36.36) | 2 (40.00) | 7 (46.67) |  |
| Multiple organ failure | 9 (11.11) | 5 (12.50) | 0 | 0 | 4 (80.00) | 0 |  |
| Arterial Hypertension | 15 (18.52) | 11 (27.50) | 4 (16.00) | 0 | 0 | 3 (20.00) |  |
| High blood sugar | 22 (27.16) | 12 (30.00） | 6 (24.00) | 3 (27.27) | 1 (20.00) | 3 (20.00) |  |
| HBV | 5 (6.17) | 4 (10.00) | 1 (4.00) | 0 | 0 | 3 (20.00) |  |
| Laboratory results | | | | | | |  |
| Abnormal white blood cell levels (<4 or >10× 109/L ) | 18 (22.22) | 8 (20.00) | 3 (12.00) | 6 (54.54) | 1 (20.00) | 6 (40.00) |  |
| Red blood cells<4 × 10^9^/L | 44 (54.32) | 28 (70.00) | 8 (32.00) | 6 (54.54) | 2 (40.00) | 11 (73.33) |  |
| Hemoglobin<105 g/L | 24 (29.63) | 17 (42.50) | 4 (16.00) | 3 (27.27) | 0 | 6 (40.00) |  |
| Platelets<100 × 10^9^/L | 18 (22.22) | 15 (37.50) | 2 (8.00) | 1 (9.10) | 0 | 4 (26.67) |  |
| Neutrophils<2× 10^9^/L | 22 (22.92) | 9 (22.50) | 5 (20.00) | 3 (27.27) | 0 | 5 (33.33) |  |
| lymphocyte<1.1 × 10^9^/L | 44 (54.32) | 23 (57.50) | 14 (56.00) | 5 (45.45) | 2 (40.00) | 7 (46.67) |  |
| RDW> 15% | 27 (33.33) | 15 (37.50) | 6 (24.00) | 6 (54.54) | 0 | 7 (46.67) |  |
| Serum LDH > 250 U/L | 45 (55.56) | 28 (70.00) | 9 (36.00) | 5 (45.45) | 3 (60.00) | 5 (33.33) |  |
| Serum β2-MG > 3 mg/L | 40 (49.38) | 23 (57.50) | 10 (40.00) | 5 (45.45) | 2 (40.00) | 11 (73.33) |  |
| *Abbreviations: RDW, Red blood cell distribution width; LDH, lactate dehydrogenase; β2-MG, β2 microglobulin.* | | | | | | |  |

| Table S3: The proportion of each marker expression in the five disease groups. | | | | | |
| --- | --- | --- | --- | --- | --- |
|  | AITL (n=40) | PTCL-NOS (n=25) | ENKTL-N (n=11) | ALCL (n=5) | T-CUS (n=15) |
| CD2 | 40 (100.00) | 24 (96.00) | 11 (100.00) | 5 (100.00) | 15 (100.00) |
| CD3 | 17 (42.50) | 13 (52.00) | 11 (100.00) | 3 (60.00) | 14 (93.33) |
| CD4 | 38 (95.00) | 16 (64.00) | 5 (45.45) | 5 (100.00) | 10 (66.67) |
| CD5 | 38 (96.00） | 18 (72.00) | 9 (81.82) | 1 (20.00) | 14 (93.33) |
| CD7 | 20 (50.00) | 19 (76.00) | 7 (63.64) | 3 (60.00) | 10 (66.67) |
| CD8 | 3 (7.50) | 8 (32.00) | 4 (36.36) | 0 | 12 (80.00) |
| CD10 | 22 (55.00) | 2 (8.00) | 0 | 0 | 2 (13.33) |
| CD16 | 1 (2.50) | 2 (8.00) | 0 | 1 (20.00) | 3 (20.00) |
| CD30 | 1 (2.50) | 2 (8.00) | 1 (9.09) | 4 (80.00) | 0 |
| CD38 | 21 (52.50) | 14 (56.00) | 4 (36.36) | 3 (60.00) | 7 (46.67) |
| CD56 | 6 (15.00) | 6 (24.00) | 9 (81.82) | 1 (20.00) | 5 (33.33) |
| CD57 | 5 (12.50) | 6 (24.00) | 8 (72.73) | 0 | 9 (60.00) |
| HLA-DR | 28 (70.00) | 17 (68.00) | 5 (45.45) | 4 (80.00) | 7 (46.67) |
| PD-1 | 35 (87.50) | 7 (28.00) | 4 (36.36) | 1 (20.00) | 6 (40.00) |
| Ki-67 | 2 (5.00) | 2 (8.00) | 0 | 3 (60.00) | 0 |

**Table S4: Reference ranges for TCR-vβ profiles.**

| *Normal Range (%)* | | | |
| --- | --- | --- | --- |
| vβ1 | 1.89-11.7 | vβ12 | 1.0-4.76 |
| vβ2 | 4.03-23.48 | vβ13.1 | 1.62-8.16 |
| vβ3 | 0.2-15.71 | vβ13.2 | 0.80-5.28 |
| vβ4 | 0.79-3.26 | vβ13.6 | 0.84-8.8 |
| vβ5.1 | 3.19-14.93 | vβ14 | 1.33-8.03 |
| vβ5.2 | 0.49-4.98 | vβ16 | 0.42-1.9 |
| vβ5.3 | 0.37-2.98 | vβ17 | 2.28-12.61 |
| vβ7.1 | 0.64-20.01 | vβ18 | 0.58-5.23 |
| vβ7.2 | 0.05-5.45 | vβ20 | 0.0-9.73 |
| vβ8 | 2.26-29.47 | vβ21.3 | 1.06-5.97 |
| vβ9 | 1.1-9.63 | vβ22 | 1.99-9.89 |
| vβ11 | 0.25-5.11 | vβ23 | 0.28-4.76 |
| *Total Coverage* | | 69.95 | |

**Table S5: Kaplan-Meier analyses of OS in PLCLs.**

|  |  | *AITL* | | | *PTCL-NOS* | | | *ENKTL-N* | | |
| --- | --- | --- | --- | --- | --- | --- | --- | --- | --- | --- |
| Markers |  | HR | 95% CI | P-Value | HR | 95% CI | P-Value | HR | 95% CI | P-Value |
| CD3 | (Positive, Negative) | 0.8005 | 0.3178 to 2.017 | 0.5668 | 3.938 | 1.181 to 13.13 | 0.0252* | NA | | |
| CD4 | (Positive, Negative) | 0.8959 | 0.1077 to 7.452 | 0.9127 | 0.7274 | 0.1987 to 2.663 | 0.6043 | 0.2709 | 0.03776 to 1.944 | 0.2215 |
| CD5 | (Positive, Negative) | NA | | | 0.6819 | 0.1572 to 2.958 | 0.5598 | 0.2371 | 0.005510 to 10.20 | 0.1553 |
| CD7 | (Positive, Negative) | 0.2464 | 0.09467 to 0.6414 | 0.006* | 1.473 | 0.4286 to 5.061 | 0.5542 | 2.302 | 0.3187 to 16.63 | 0.4552 |
| CD8 | (Positive, Negative) | 0.8368 | 0.2118 to 3.306 | 0.8021 | 4.284 | 1.105 to 16.61 | 0.0076* | 1.666 | 0.2201 to 12.61 | 0.6051 |
| CD10 | (Positive, Negative) | 0.7342 | 0.2907 to 1.854 | 0.5017 | 1.946 | 0.2773 to 13.65 | 0.3769 | NA | | |
| CD16 | (Positive, Negative) | 3.46 | 0.09422 to 127.1 | 0.187 | 3.158 | 0.1027 to 97.11 | 0.2267 | NA | | |
| CD30 | (Positive, Negative) | NA | | | 0.7867 | 0.1215 to 5.095 | 0.816 | 0.3208 | 0.01521 to 6.766 | 0.4725 |
| CD38 | (Positive, Negative) | 3.473 | 1.341 to 8.991 | 0.0168* | 1.821 | 0.5582 to 5.938 | 0.3248 | 2.245 | 0.2688 to 18.75 | 0.4039 |
| CD56 | (Positive, Negative) | 2.031 | 0.4984 to 8.278 | 0.1906 | 1.661 | 0.4288 to 6.435 | 0.4056 | 0.1737 | 0.01824 to 1.654 | 0.0105* |
| CD57 | (Positive, Negative) | 1.065 | 0.3437 to 3.302 | 0.9798 | 1.396 | 0.3798 to 5.133 | 0.5841 | 0.3896 | 0.04397 to 3.453 | 0.3248 |
| HLA-DR | (Positive, Negative) | 2.431 | 0.8151 to 7.250 | 0.2026 | 0.5503 | 0.1146 to 2.643 | 0.3601 | 8.011 | 0.8866 to 72.38 | 0.0288 |
| Ki-67 | (Positive, Negative) | 7.553 | 0.4202 to 135.8 | 0.0001* | 3.108 | 0.2911 to 33.18 | 0.118 | NA | | |
| PD-1 | (Positive, Negative) | 0.892 | 0.2458 to 3.237 | 0.8513 | 0.2245 | 0.06242 to 0.8072 | 0.1133 | 1.946 | 0.2457 to 15.42 | 0.4965 |
| *NA: not available; *: P value less than or equal to 0.05.* | | | | | | | | | | |

**Table S6: Kaplan-Meier analyses of PFS in PLCL.**

|  |  | *AITL* | | | *PTCL-NOS* | | | *ENKTL-N* | | |
| --- | --- | --- | --- | --- | --- | --- | --- | --- | --- | --- |
| Markers |  | HR | 95% CI | P-Value | HR | 95% CI | P-Value | HR | 95% CI | P-Value |
| CD3 | (Positive, Negative) | 1.183 | 0.4693 to 2.980 |  | 2.541 | 0.7789 to 8.287 | 0.21 | NA | | |
| CD4 | (Positive, Negative) | 0.4742 | 0.02761 to 8.143 | 0.4485 | 0.8443 | 0.2392 to 2.980 | 0.7824 | 0.291 | 0.04083 to 2.076 | 0.2513 |
| CD5 | (Positive, Negative) | NA | | | 0.7199 | 0.1698 to 3.052 | 0.6186 | 0.237 | 0.005510 to 10.20 | 0.1553 |
| CD7 | (Positive, Negative) | 0.338 | 0.1339 to 0.8531 | 0.0239* | 1.306 | 0.3711 to 4.594 | 0.6868 | 2.088 | 0.2847 to 15.31 | 0.5106 |
| CD8 | (Positive, Negative) | 0.7109 | 0.1946 to 2.596 | 0.6307 | 2.19 | 0.6365 to 7.537 | 0.1762 | 1.545 | 0.2077 to 11.49 | 0.6585 |
| CD10 | (Positive, Negative) | 0.8573 | 0.3368 to 2.182 | 0.7373 | 1.506 | 0.2577 to 8.805 | 0.589 | NA | | |
| CD16 | (Positive, Negative) | 3.46 | 0.09422 to 127.1 | 0.187 | 3.158 | 0.1027 to 97.11 | 0.2267 | NA | | |
| CD30 | (Positive, Negative) | NA | | | 0.7867 | 0.1215 to 5.095 | 0.816 | 0.321 | 0.01521 to 6.766 | 0.4648 |
| CD38 | (Positive, Negative) | 2.516 | 0.9987 to 6.340 | 0.0286* | 1.603 | 0.4912 to 5.234 | 0.4291 | 2.124 | 0.2599 to 17.36 | 0.4386 |
| CD56 | (Positive, Negative) | 1.873 | 0.4792 to 7.322 | 0.2499 | 1.661 | 0.4288 to 6.435 | 0.4056 | 0.098 | 0.009514 to 1.018 | 0.0219* |
| CD57 | (Positive, Negative) | 1.062 | 0.3429 to 3.288 | 0.9091 | 0.906 | 0.2692 to 3.049 | 0.8707 | 0.413 | 0.04788 to 3.562 | 0.3571 |
| HLA-DR | (Positive, Negative) | 2.579 | 0.8793 to 7.563 | 0.1737 | 0.6322 | 0.1409 to 2.838 | 0.487 | 4.388 | 0.5603 to 34.36 | 0.0327* |
| Ki-67 | (Positive, Negative) | 1.967 | 0.2796 to 13.85 | 0.3554 | 2.377 | 0.2872 to 19.68 | 0.2401 | NA | | |
| PD-1 | (Positive, Negative) | 0.8474 | 0.2281 to 3.148 | 0.7871 | 0.3081 | 0.07798 to 1.217 | 0.2219 | 0.559 | 0.05797 to 5.396 | 0.6301 |
| *NA: not available; *: P value less than or equal to 0.05.* | | | | | | | | | | |

**Table S7: Univariate and multivariate Cox regression analyses of OS in AITL.**

| *OS* |  |  | *Univariate analysis* | | | *Multivariate analysis* | | |
| --- | --- | --- | --- | --- | --- | --- | --- | --- |
|  | Factor |  | HR | 95% CI | P-Value | HR | 95% CI | P-Value |
|  | Age | (>60,≤60) | 3.236 | 1.209 to 8.665 | 0.0062 | NS | | |
|  | Sex | (male, female) | 0.9499 | 0.3661 to 2.464 | 0.9086 |  |  |  |
|  | IPI | (High risk, Medium risk) | 2.14 | 0.8215 to 5.576 | 0.0361 | 3.906 | 1.407 to 10.870 | 0.009 |
|  | B symptoms | (Yes, No) | 0.9044 | 0.3465 to 2.361 | 0.7863 |  |  |  |
|  | LDH | (>245 U/L, ≤245 U/L) | 2.559 | 0.9821 to 6.666 | 0.0311 | NS | | |
|  | β2-MG | (>3 mg/L, ≤3 mg/L) | 2.523 | 0.9381 to 6.787 | 0.0384 | NS | | |
|  | CD7 | (Positive, Negative | 0.2464 | 0.09467 to 0.6414 | 0.006 | 0.14 | 0.044 to 0.511 | 0.004 |
|  | CD38 | (Positive, Negative | 3.473 | 1.341 to 8.991 | 0.0168 | 3.984 | 1.458 to 14.706 | 0.014 |
|  | KI67 | (Positive, Negative | 7.553 | 0.4202 to 135.8 | 0.0001 | NS | | |
| *NS, no significance.* | | | | | | | | |

**Table S8: Univariate and multivariate Cox regression analyses of PFS in AITL.**

|  | | | | | | | | |
| --- | --- | --- | --- | --- | --- | --- | --- | --- |
| *PFS* |  |  | Univariate analysis | | | Multivariate analysis | | |
|  | Factor |  | HR | 95% CI | P-Value | HR | 95% CI | P-Value |
|  | Age | (>60,≤60) | 2.508 | 0.8472 to 7.426 | 0.0371 | NS | | |
|  | Sex | (male, female) | 1.081 | 0.4224 to 2.769 | 0.4501 |  |  |  |
|  | IPI | (High risk, Medium risk) | 2.334 | 0.9099 to 5.988 | 0.0115 | 3.521 | 1.157 to 10.750 | 0.027 |
|  | B symptoms | (Yes, No) | 1.015 | 0.3858 to 2.670 | 0.5497 |  |  |  |
|  | LDH | (>245 U/L, ≤245 U/L) | 1.01 | 0.3568 to 2.862 | 0.698 |  |  |  |
|  | β2-MG | (>3 mg/L, ≤3 mg/L) | 1.361 | 0.5236 to 3.536 | 0.2053 |  |  |  |
|  | CD7 | (Positive, Negative | 0.338 | 0.1339 to 0.8531 | 0.0239 | 0.098 | 0.023 to 0.412 | 0.002 |
|  | CD38 | (Positive, Negative | 2.516 | 0.9987 to 6.340 | 0.0286 | NS | | |
| *NS, no significance.* | | | | | | | | |

**Table S9: Univariate and multivariate Cox regression analyses of OS in PLCL-NOS.**

| *OS* |  |  | *Univariate analysis* | | | *Multivariate analysis* | | |
| --- | --- | --- | --- | --- | --- | --- | --- | --- |
|  | Factor |  | HR | 95% CI | P-Value | HR | 95% CI | P-Value |
|  | Age | (>60,≤60) | 5.236 | 1.396 to 19.64 | 0.0141 | NS | | |
|  | Sex | (male, female) | 0.4779 | 0.1459 to 1.566 | 0.2191 |  |  |  |
|  | PIT | (High risk, Medium risk) | 7.254 | 1.987 to 26.48 | 0.0022 | NS | | |
|  | B symptoms | (Yes, No) | 1.381 | 0.3767 to 5.060 | 0.5951 |  |  |  |
|  | LDH | (>245 U/L, ≤245 U/L) | 0.5287 | 0.1347 to 2.076 | 0.3611 |  |  |  |
|  | β2-MG | (>3 mg/L, ≤3 mg/L) | 1.571 | 0.4545 to 5.427 | 0.472 |  |  |  |
|  | CD3 | (Positive, Negative | 3.938 | 1.181 to 13.13 | 0.0252 | NS | | |
|  | CD8 | (Positive, Negative | 4.284 | 1.105 to 16.61 | 0.0076 | NS | | |
| *NS, no significance.* | | | | | | | |  |

**Table S10: Univariate and multivariate Cox regression analyses of OS in ENKTL-N.**

| *OS* |  |  | *Univariate analysis* | | | *Multivariate analysis* | | |
| --- | --- | --- | --- | --- | --- | --- | --- | --- |
|  | Factor |  | HR | 95% CI | P-Value | HR | 95% CI | P-Value |
|  | Age | (>60,≤60) | 10.12 | 0.2670 to 383.4 | 0.212 |  |  |  |
|  | Sex | (male,female) | 0.4867 | 0.06110 to 3.877 | 0.4061 |  |  |  |
|  | PINK | (High risk,Medium risk) | 1.956 | 0.2663 to 14.36 | 0.5097 |  |  |  |
|  | B symptoms | (Yes, No) | 11.06 | 1.011 to 120.9 | 0.0071 | NS | | |
|  | LDH | (>245 U/L, ≤245 U/L) | 1 | 0.05009 to 19.96 | >0.9999 |  |  |  |
|  | β2-MG | (>3 mg/L, ≤3 mg/L) | 3.016 | 0.3351 to 27.14 | 0.3248 |  |  |  |
|  | CD56 | (Positive, Negative | 0.1737 | 0.01824 to 1.654 | 0.0105 | NS | | |
| *NS, no significance.* | | | | | | | |  |

**Table S11: Univariate and multivariate Cox regression analyses of PFS in ENKTL-N.**

| *PFS* |  |  | *Univariate analysis* | | | *Multivariate analysis* | | |
| --- | --- | --- | --- | --- | --- | --- | --- | --- |
|  | Factor |  | HR | 95% CI | P-Value | HR | 95% CI | P-Value |
|  | Age | (>60,≤60) | 1.176 | 0.1602 to 8.628 | 0.8735 |  |  |  |
|  | Sex | (male,female) | 0.5416 | 0.06952 to 4.219 | 0.5331 |  |  |  |
|  | PINK | (High risk,Medium risk) | 1.904 | 0.2548 to 14.22 | 0.5693 |  |  |  |
|  | B symptoms | (Yes, No) | 5.751 | 0.7296 to 45.34 | 0.0258 | NS | | |
|  | LDH | (>245 U/L, ≤245 U/L) | 1.103 | 0.09589 to 12.69 | 0.7681 |  |  |  |
|  | β2-MG | (>3 mg/L, ≤3 mg/L) | 2.421 | 0.2807 to 20.88 | 0.3571 |  |  |  |
|  | CD56 | (Positive, Negative | 0.0984 | 0.009514 to 1.018 | 0.0219 | NS | | |
|  | HLA-DR | (Positive, Negative | 4.388 | 0.5603 to 34.36 | 0.0327 | NS | | |
| *NS, no significance.* | | | | | | | |  |
